# The Yellow Fever Commission

**Published:** 1878-11

**Authors:** 


### THE YELLOW FEVER COMMISSION.

It will be remembered that in September last Dr. Woodworth, Surgeon General of Marine Hospital Service, appointed a commission to investigate the origin and behavior of yellow fever poison. Secular papers now announce the termination of their labors, and the conclusions arrived at. Nothing new seems to have been discovered, and no startling theory is to be promulgated—the same old doctrine is to be continued. Newspapers say the commission declare yellow fever exotic; that it was certainly imported into New Orleans the past summer, and carried up the river. Further, that the only safety to this country lies in efficient national quarantine.

Well, this importation and transportation theory has been adopted by a majority of those concerned in such matters for a century past. Certainly the creator of this commission advocated the same theory in a recent publication, and doubtless his appointees were known to agree with him. Commissions, therefore, appointed in this way, may, from honest convictions, reflect the opinions of the creator.

Until their report has been made, of course, the reasons for their opinions are unknown; but certain facts, of which we presume they were informed, will subject their reputed decision to just criticism.

In the next number of this journal, with the commission's report before us, we shall make such comments as the importance of this subject demands. In the meantime, we must be allowed to indulge and express the hope that the people of yellow fever districts will not make up their minds to rely for protection against the disease, on "national quarantine," however strictly enforced by the Government of the United States. Thousands of valuable lives have been lost by relying upon quarantine and remaining where they imbibe daily the most malignant yellow fever malaria, till mortally stricken with the disease. Not only so, but this dependence upon the prevention of importation destroys all effort to abate prolific cause of the fever in their immediate neighborhood, such as marshes, pools, etc., filled by overflow of streams.

Common decency and every feeling of humanity is outraged by the barbarous system of inland quarantine practiced against refugees from infected districts. None but panic-stricken, selfish alarmists can read with composure the treatment of homeless wanderers in many parts of the country recently.

If, as report has it, the expenses of the commission are paid by contribution, prompted by the humane instincts of a philanthropic female, and the work of investigation, undertaken at the risk of life by the friends of our race, we are constrained to cry out, in view of the reputed report, " Save us from our friends."

